# Template-directed atomically precise self-organization of perfectly ordered parallel cerium silicide nanowire arrays on Si(110)-16 × 2 surfaces

**DOI:** 10.1186/1556-276X-8-458

**Published:** 2013-11-05

**Authors:** Ie-Hong Hong, Yung-Cheng Liao, Yung-Feng Tsai

**Affiliations:** 1 Department of Electrophysics, National Chiayi University, Chiayi 60004, Taiwan; 2 Institute of Optoelectronics and Solid State Electronics, National Chiayi University, Chiayi 60004, Taiwan

**Keywords:** Self-organization, Nanowires, Ce silicide, Si(110), Scanning tunneling microscopy

## Abstract

The perfectly ordered parallel arrays of periodic Ce silicide nanowires can self-organize with atomic precision on single-domain Si(110)-16 × 2 surfaces. The growth evolution of self-ordered parallel Ce silicide nanowire arrays is investigated over a broad range of Ce coverages on single-domain Si(110)-16 × 2 surfaces by scanning tunneling microscopy (STM). Three different types of well-ordered parallel arrays, consisting of uniformly spaced and atomically identical Ce silicide nanowires, are self-organized through the heteroepitaxial growth of Ce silicides on a long-range grating-like 16 × 2 reconstruction at the deposition of various Ce coverages. Each atomically precise Ce silicide nanowire consists of a bundle of chains and rows with different atomic structures. The atomic-resolution dual-polarity STM images reveal that the interchain coupling leads to the formation of the registry-aligned chain bundles within individual Ce silicide nanowire. The nanowire width and the interchain coupling can be adjusted systematically by varying the Ce coverage on a Si(110) surface. This natural template-directed self-organization of perfectly regular parallel nanowire arrays allows for the precise control of the feature size and positions within ±0.2 nm over a large area. Thus, it is a promising route to produce parallel nanowire arrays in a straightforward, low-cost, high-throughput process.

## Background

One-dimensional (1D) nanowires (NWs) have attracted significant attention in condensed matter physics and nanoelectronics because they exhibit peculiar properties due to many-body interactions in a 1D system [1,2]. In particular, epitaxial rare-earth silicide (RES; RE = Y [3], Gd [4,5], Dy [5,6], and Er [5,7]) NWs self-assembled on flat Si(100)-2 × 1 surfaces have been intensively studied by utilizing the anisotropic lattice mismatch between the hexagonal RES and the Si(100) surfaces. The metallic RES NWs with high aspect ratios have potential applications as interconnects in nanoelectronic devices because of their high conductivity, extremely low Schottky barrier height on n-type Si, perfect single crystalline, and atomically sharp interfaces with Si substrates. Moreover, these RES NWs also exhibit highly anisotropic band structures along the NW direction [4,6]; they are another prototype of 1D electron systems.

Among a large variety of RES compounds, cerium silicide (CeSi_*x*_) compounds (0.8 ≤ *x* ≤ 5.0) have attracted widespread interest owing to their several peculiar physical properties, such as intermediate valency, Kondo lattice, heavy fermion superconductivity, anisotropic transport, and magnetic ordering behavior, which originate from the interplay between the strong correlations of Ce 4*f* electrons and the hybridization of 4*f* electrons and conduction electrons [8-14]. Additionally, Ce-doped Si films have been found to exhibit various magnetic phenomena below 100 K, such as superparamagnetism, spin-glass behavior, and giant magnetoresistance [15,16]. Furthermore, Si substrates have been regarded as ideal hosts for spin transport because of their long spin relaxation time due to a weak spin-orbit interaction, which leads to a long spin diffusion length in spintronic devices [17,18]. Therefore, CeSi_*x*_ NWs grown epitaxially on Si surfaces can become a promising 1D nanomaterial for Si-based spintronic applications. In this regard, there is an ongoing interest in the self-organization of CeSi_*x*_ NWs on Si surfaces [19-21].

To understand precisely the exotic 1D physics of electrons in CeSi_*x*_ NWs and to explore its utility for spintronic applications, it is essential to grow a perfectly ordered parallel array of periodic and atomically identical CeSi_*x*_ NWs over a large area, allowing the accurate investigation of their detailed chemical, electronic, and magnetic structures by photoemission spectroscopy and magnetic circular dichroism [22]. However, so far, no large-area (>1 × 1 μm^2^), well-regular parallel CeSi_*x*_ NW arrays with uniform distribution and identical dimension can be formed on flat and vicinal Si(100) surfaces.

Recently, we have demonstrated that RE metals (e.g., Gd, Ce, and Er) can be self-organized to form a mesoscopically ordered parallel RES NW array on single-domain Si(110)-16 × 2 surfaces [23-25]. These parallel-aligned and unidirectional RES NWs exhibit identical sizes, periodic positions, large aspect ratios (length >1 μm, width ≤5 nm) exceeding 300, and ultra-high integration density up to 10^4^ NWs/μm^2^. Such large-area self-ordered growths of massively parallel RES NW arrays on Si(110) surfaces can open the possibility for wafer-scale integration into nanoelectronic devices combining the well-established Si(110)-based integrated-circuit technology [26-28] with the exotic 1D physical properties of RES NWs.

To date, there is little knowledge of this template-directed 1D self-organization process that leads to the formation of well-ordered parallel RES NW arrays on single-domain Si(110)-16 × 2 surfaces. In this article, we have investigated the growth evolutions of CeSi_*x*_ NWs on Si(110) surfaces over a wide range (1 to 9 monolayers (ML)) of Ce coverage by scanning tunneling microscopy (STM). Our comprehensive study provides a detailed understanding of the 1D self-organization mechanism of perfectly ordered parallel arrays consisting of periodic and atomically identical CeSi_*x*_ NWs on single-domain Si(110)-16 × 2 surfaces.

## Methods

**Figure 1:**
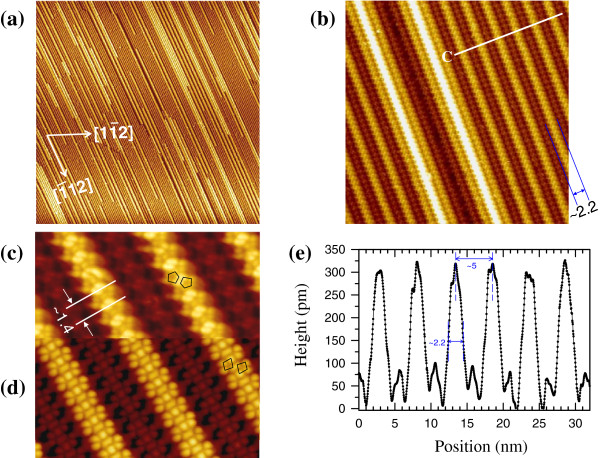
**STM images and topography profile of the atomically clean Si(110)-16 × 2 surface.** A series of STM topographic images of the atomically clean Si(110)-16 × 2 surface taken at different magnifications: **(a)** 850 × 850 nm^2^ (bias voltage *V*_b_ = +2.0 V, tunneling current *I*_t_ = 0.1 nA), **(b)** 70 × 70 nm^2^, and **(c, d)** dual-polarity STM images (25 × 15 nm^2^) acquired at +1.6 and -1.6 V, respectively, and at 20 pA. **(e)** Topography profile C across the up-and-down terraces of the 16 × 2 superstructure along the white lines indicated in (b).

Our experiments were performed in an ultra-high vacuum, variable-temperature STM system (Omicron Nanotechnology GmbH, Taunusstein, Germany) with a base pressure of less than 3.0 × 10^-11^ mbar. An n-type P-doped Si(110) surface with a resistivity of about 10 Ω cm was cleaned by well-established annealing procedures [25,29,30]. An atomically clean single-domain Si(110)-16 × 2 surface was confirmed by STM observation (Figure 1). Different parallel CeSi_*x*_ NW arrays were produced by depositing high-purity (99.95%) Ce metals with coverages ranging from 1 to 9 ML (1 ML = 9.59 × 10^14^ atoms/cm^2^) onto a single-domain Si(110)-16 × 2 surface at 675 K with a deposition rate of 0.15 ML/min and subsequently annealed at 875 K for 20 min. The growth temperature cannot be higher than 675 K; otherwise, a large amount of Ce clusters will be formed [20,21]. Ce metals were evaporated from an electron-beam evaporator with an internal flux meter; their deposition coverage was determined *in situ* by a quartz crystal thickness monitor with an accuracy of 20%. The sample temperature was measured using an infrared pyrometer with an uncertainty of ± 30 K. The chamber pressure remained below 1.0 × 10^-9^ mbar during evaporation. The STM measurements were acquired at 300 K using electrochemically etched nickel tips.

## Results and discussion

### Morphology and structure of the atomically clean Si(110)-16 × 2 surface

Figure 1a represents a typical large-scale (850 × 850 nm^2^) STM image of an atomically clean Si(110)-16 × 2 surface. The parallel up-and-down terraces of the 16 × 2 reconstruction have a huge area exceeding 2 × 2 μm^2^. Such uniform grating-like terraces over a large region can be used as a perfect template for the large-scale self-organization of a well-ordered parallel silicide NW array. In Figure 1b, a magnified image (70 × 70 nm^2^) clearly shows zigzag chains formed on the upper and lower terraces; the period of zigzag chains is 1.4 ± 0.2 nm [31,32], indicated in Figure 1c. Additionally, two highest terraces with the white contrast are seen together with the pairs of the upper (bright) and lower (dark) terraces. The set of terraces with dark, bright, and white contrasts, due to the vertical height difference, forms the (17 15 1) vicinal facet and often coexist in 16 × 2 reconstruction [33]. Figure 1c,d depicts the empty-state and filled-state STM images of this 16 × 2 reconstruction at atomic resolution. A pair of Si pentagons/tetramers forming zigzag chains in the upper and lower terraces is clearly resolved, as marked by two schematic pentagons/tetramers on the upper terraces in the empty-state/filled-state STM images, consistent with previous result [32]. Figure 1e displays the cross-sectional profile across the up-and-down terraces of the 16 × 2 reconstruction along the line scan C in Figure 1b. The typical width and average height of these periodic upper terraces are 2.2 ± 0.2 nm and 300 ± 10 pm, respectively, and the periodicity (i.e., the pitch) of the uniformly spaced upper terraces is 5.0 ± 0.1 nm. These nanoscale sizes of upper and lower terraces on the Si(110) surface can make the template-directed self-organization with atomic precision.

### Coverage-dependent morphologies and structures of CeSi_*x*_ NWs

**Figure 2:**
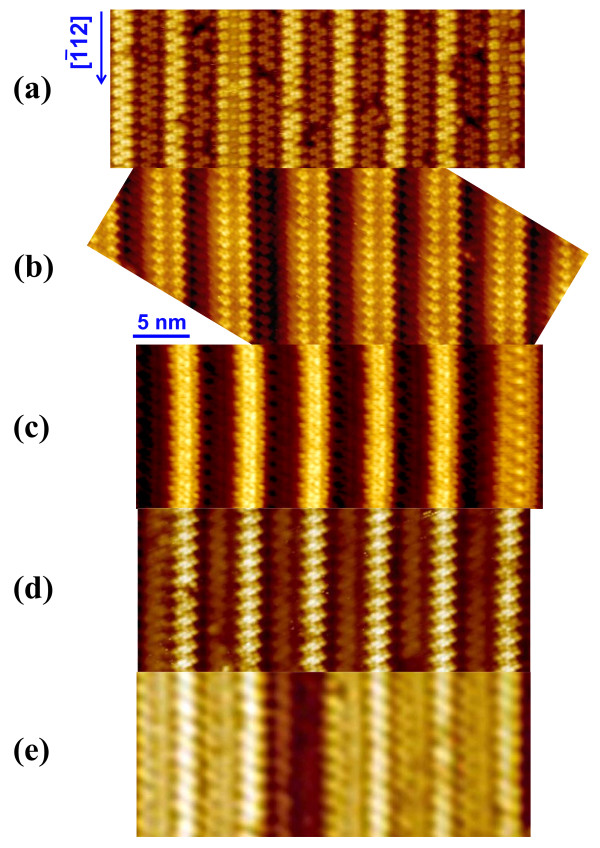
**Growth evolution of epitaxial CeSi**_***x ***_**NWs on the Si(110) surfaces for different Ce coverages.** The STM morphologies of CeSi_*x*_ NWs grown on the Si(110) surfaces for different Ce coverages: **(a)** 1 ML, **(b)** 3 ML, **(c)** 5 ML, **(d)** 6 ML, and **(e)** 9 ML. The image scale of 5 nm is indicated by a bar.

Figure 2 shows a series of STM topographic images of CeSi_*x*_ NWs self-organized on the Si(110) surface for different Ce coverages. At the initial growth stage (i.e., 1-ML Ce deposition) in Figure 2a, besides the pristine upper and lower Si terraces with the zigzag chains of pentagon pair, we can obviously see that two straight and robust CeSi_*x*_ NWs are formed on the upper Si terraces due to the preferential reactivity of Ce atoms with Si pentagon pair on the upper terraces, consistent with the formation of GdSi_*x*_/ErSi_*x*_ NWs on the upper terraces of Si(110) [23,25]. However, this observation is in contrast to prior results that show the formation of NWs on the step edge of vicinal Si surfaces [34]. As the coverage increases to 3 ML, the CeSi_*x*_ NWs become denser and are regularly distributed. Moreover, these parallel-aligned NWs are uniform in height and width over their length. With the increase of Ce coverage (Figure 2c,d,e), different types of CeSi_*x*_ NWs with different chain structures are formed. As demonstrated in Figure 2, most NWs are always atomically identical and homogeneously positioned when the coverage is above 3 ML. Because the structural evolution of CeSi_*x*_ NWs for different coverages can be roughly divided into three various growth stages (i.e., at the Ce coverages of 3, 6, and 9 ML), we investigate in detail the coverage-dependent growth behaviors of the self-ordered CeSi_*x*_ NWs at these three different growth stages in the following.

### 3-ML Ce deposition

**Figure 3:**
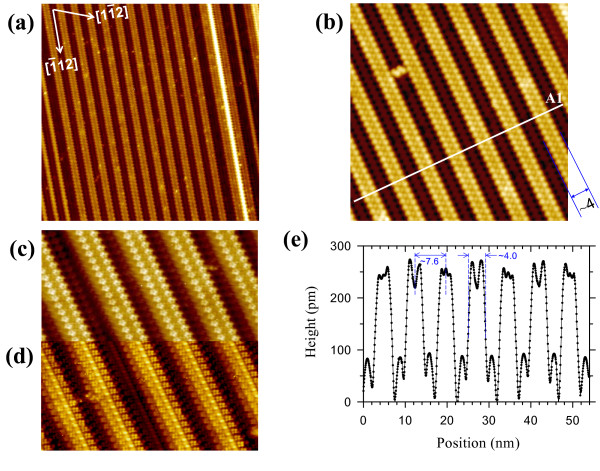
**STM images and topography profile of the parallel 3-NW array on the Si(110) surface.** A series of different magnified STM topographic images of the parallel-aligned and periodic 3-NWs: **(a)** 120 × 120 nm^2^ (*V*_b_ = +2.0 V, *I*_t_ = 60 pA), **(b)** 50 × 50 nm^2^, and **(c**, **d)** dual-polarity STM images (40 × 20 nm^2^) acquired at +1.2 and -1.2 V, respectively, and at 30 pA. **(e)** Cross-sectional profile A1 across parallel-aligned 3-NWs along the white line indicated in (b).

Figure 3a,b,c,d shows a sequence of different magnified STM topographic images of the parallel CeSi_*x*_ NW array obtained by depositing 3-ML Ce on the Si(110) surface; these NWs are labeled as 3-NWs. As clearly seen in Figure 3a,b,c,d, the parallel-aligned, very straight, and nearly defect-free NWs are elongated along the [$\bar{1}\;1\;2$] direction and show a periodic pitch. These parallel NWs are atomically identical to one another over a huge area exceeding 1 × 1 μm^2^. As shown in the lower left region of Figure 3a, three pristine upper Si terraces are adjacent to the parallel 3-NWs and still show the pitch of 5.0 ± 0.1 nm, indicating that the 3-NWs are indeed formed on the upper Si terraces rather than on the lower Si terraces, consistent with the observation in Figure 2a. In Figure 3b, each 3-NW consists of double bead chains separated by a bean chain. Each bead chain is composed of round protrusions with a diameter of 1.4 ± 0.1 nm. The diameter and the periodicity of protrusions in a bean chain are 1.2 ± 0.1 and 1.4 ± 0.1 nm, respectively. The substrate between neighboring 3-NWs still shows a zigzag-like chain structure, similar to the appearance of the lower Si terraces.

Figure 3c,d shows an enlarged portion of the image in Figure 3b, recorded at two bias voltages *V*_b_ = +1.2 and -1.2 V, respectively. In the dual-polarity images, the parallel, registry-aligned NWs are well resolved. The empty-state image clearly shows that the rounded protrusions of the double bead chains in Figure 3b convert to pentagons and the bean chain becomes a trench. However, the pentagons in the left and right bead chains are oppositely oriented, similar to the orientation of the Si pentagon pair (Figure 1c). In the filled-state image, each 3-NW appears to comprise two chains of tetramers with the opposite orientation at both sides, similar to the orientation of the Si tetramer pair (Figure 1d), and a bean chain at the middle of the NW. Moreover, the contrast of these double tetramer chains is lower than that of the bean chain. Notice that the dark trench in Figure 3c inverts to the bright bean chains in Figure 3d when the bias polarity is reversed. The polarity dependence of these STM images clearly reveals that each 3-NW consists of a bundle of three chain structures with a charge modulation of alternating filled and empty states, indicating a pronounced ionicity of the chains [35]. These results strongly suggest that the Si pentagon/tetramer pair on the upper terraces of the 16 × 2 reconstruction (Figure 1c,d) is split into two individual Si pentagons/tetramers upon Ce adsorption due to the preferential reactivity of Ce atoms with the Si pentagon pair on the upper terraces (Figure 2a), thereby leading to the formation of a bean chain at the middle of the 3-NWs.

Figure 3e plots the cross-sectional profiles of the line scan A1 across the parallel 3-NWs in Figure 3b. The average width of the 3-NWs is 4.0 ± 0.1 nm, which is about two times the width of the Si terrace (i.e., 2.2 ± 0.2 nm) as explained above. Also due to the strong chemical interaction of Ce atoms and the Si pentagon pair on the upper terraces, the typical NW height is decreased to 250 ± 10 pm, lower than the height of the upper Si terraces (i.e., 300 ± 10 pm). The periodicity of this parallel NW array is 7.6 ± 0.2 nm. However, the height of the zigzag chains on the substrate (i.e., 90 ± 10 pm) is almost identical to that of the lower Si terraces (i.e., 90 ± 15 pm), indicating that the morphology of the pristine lower Si terraces is nearly unchanged upon Ce deposition. These results support that most Ce atoms are preferentially adsorbed on the upper Si terraces. Therefore, the self-organization of this parallel array of uniformly spaced 3-NWs on the Si(110) surface is mainly driven by the heteroepitaxial growth of CeSi_*x*_ on these periodic upper terraces of the Si(110)-16 × 2 superstructure.

The dimensions of the 3-NWs are similar to those of the GdSi_*x*_ NWs [23]. The origin of this similarity is explained in the identical 1D building block structure of these systems, i.e., the upper Si terraces. Although the empty-state image of the 3-NWs (Figure 3a,b,c) is very similar to that of the GdSi_*x*_ NWs, the filled-state image of the 3-NWs (Figure 3d) is different from that of the GdSi_*x*_ NWs, which may be attributed to the different electronic configurations of Ce (4*f*^ 1^5*d*^1^6*s*^2^) and Gd (4*f*^ 7^5*d*^1^6*s*^2^) that lead to different electron interactions on the Si(110) surface.

### 6-ML Ce deposition

**Figure 4:**
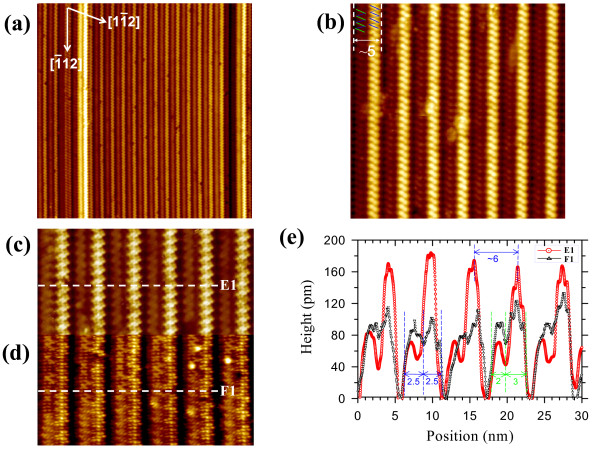
**STM images and topography profile of the parallel 6-NW array on the Si(110) surface.** A series of different magnified STM topographic images of the parallel-aligned and periodic 6-NWs: **(a)** 120 × 120 nm^2^ (*V*_b_ = +2.5 V, *I*_t_ = 60 pA), **(b)** 45 × 45 nm^2^ (*V*_b_ = 2.0 V, *I*_t_ = 40 pA), and **(c**, **d)** dual-polarity STM images (35 × 18 nm^2^) acquired at +1.5 and -1.5 V, respectively, and at 40 pA. Two zigzag lines are sketched on a 6-NW in (b) to indicate the formation of double zigzag chains in a 6-NW. **(e)** Cross-sectional profiles of E1 and F1 across the empty-state and filled-state images of parallel-aligned 6-NWs along the white dashed lines indicated in (c) and (d), respectively.

Figure 4a,b,c,d shows various magnified STM topographic images of the parallel CeSi_*x*_ NW array obtained by depositing 6-ML Ce on the Si(110) surface, which are labeled as 6-NWs. As clearly seen in Figure 4a,b, each 6-NW consists of double nonequivalent zigzag chains (indicated by two zigzag lines in Figure 4b) with different apparent heights. The left-right asymmetry observed in the height profile of the 6-NWs (Figure 4e) is different from the symmetrical morphology of the upper and lower terraces of the 16 × 2 superstructure (Figure 1e). These 6-NWs are very straight and parallel-aligned along the [$\bar{1}\;1\;2$] direction, extending over an extremely long length exceeding 1.5 μm [24]. These NWs thus possess an extraordinarily high aspect ratio beyond 300. This massively parallel NW array also shows a regular periodicity and a high integration density. Moreover, these parallel-aligned NWs are essentially identical to one another over the entire macroscopic area of the Si(110) surface. However, a few vacancy defects are present in the 6-NWs.

Figure 4c,d shows the dual-polarity STM images of an enlarged area of the parallel 6-NW array in Figure 4b, recorded at *V*_b_ = +1.5 and -1.5 V, respectively. The empty-state image clearly shows a set of double zigzag chains with noticeably different apparent heights in each 6-NW. The right zigzag chains appear much higher than the left chains. However, the filled-state image shows that the individual 6-NW consists of two linear rows with distinct atomic arrangements, and the right linear rows are also higher than the left rows. The brightest large round protrusions in Figure 4d are extra Ce clusters. The dual-polarity STM images evidently show that the 6-NWs are registry-aligned and that each 6-NW indeed comprises a bundle of double chain structures with different morphologies and different atomic structures.

Figure 4e plots the superposition of the cross-sectional profiles of both line scans E1 and F1 across the empty-state and filled-state images of the parallel 6-NWs in Figure 4c,d. As clearly revealed in Figure 4e, all the parallel-aligned 6-NWs have an identical width of 5.0 ± 0.2 nm and an equal pitch of 6.0 ± 0.2 nm in both the empty-state and filled-state images. However, the widths of the left and right zigzag chains are 2.0 ± 0.2 and 3.0 ± 0.2 nm, respectively, while the double linear rows are equal to 2.5 ± 0.2 nm, close to the widths of the upper and lower terraces of the Si(110)-16 × 2 reconstruction (i.e., 2.2 ± 0.2 nm). The heights of the left and right zigzag chains are 70 ± 10 and 170 ± 10 pm, respectively, whereas the heights of the left and right linear rows are 90 ± 10 and 120 ± 10 pm, respectively.

The right chain height of 6-NW is much lower than the height of 3-NW, indicating that there could be an inward vertical relaxation of Ce atoms upon additional Ce adsorption, but the left chain height of 6-NW is slightly smaller than the height of the pristine lower Si terraces, suggesting that the left chain originates from the epitaxial growth of CeSi_*x*_ on the lower terrace and also may contain an inward vertical relaxation. In Figure 4e, the topographic maxima of the double zigzag chains in the empty-state image and the double linear rows in the filled-state image are localized in the same spatial area (i.e., the right chains/rows). The spatial coincidence of the empty and filled states indicates that the 6-NWs may exhibit a covalent character. The results of Figure 4 strongly suggest that Ce atoms nucleated concurrently along the upper and lower terraces of the Si(110) surface to form CeSi_*x*_ NWs consisting of double chain rows with different apparent heights.

### 9-ML Ce deposition

**Figure 5:**
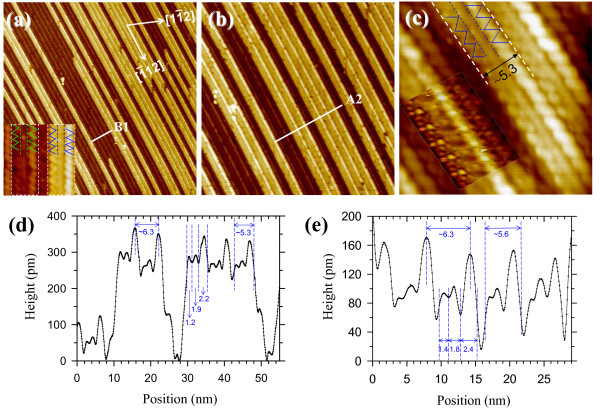
**STM images and topography profile of the parallel 9-NW array on the Si(110) surface.** A series of different magnified STM topographic images of the parallel-aligned and periodic 9-NWs: **(a)** 250 × 250 nm^2^ (*V*_b_ = +2.5 V, *I*_t_ = 80 pA), **(b)** 125 × 125 nm^2^, and **(c)** 25 × 25 nm^2^ (*V*_b_ = +2.0 V, *I*_t_ = 60 pA). Two zigzag lines and two parallel dashed lines are sketched at both sides and the middle of a 9-NW in (a) and (c) to indicate the formation of two zigzag chains and one linear row in a 9-NW. **(d)** Cross-sectional profile of A2 across parallel-aligned 9-NWs along the white lines indicated in (b). **(e)** Cross-sectional profile of B1 across the substrate along the white lines indicated in (a). The inset of (a) displays the zoom-in STM image of the substrate. The inset of (c) shows the filled-state image of the 9-NW at *V*_b_ = -1.5 V, *I*_t_ = 20 pA.

Figure 5a,b,c shows various magnified STM topographic images of the parallel CeSi_*x*_ NW array obtained by depositing 9-ML Ce on the Si(110) surface, which are labeled as 9-NWs. As shown in Figure 5a,b, these 9-NWs are still straight and parallel-aligned along the [$\bar{1}\;1\;2$] direction, with their length exceeding 1 μm. However, the NW density is not high, which may be due to the insufficient Ce amount for this growth stage. Figure 5c,d clearly depicts that each 9-NW exhibits a bundle of two nonequivalent zigzag chains (indicated by two zigzag lines) with different widths/heights of 1.2 ± 0.2/0.28 ± 0.02 nm (left) and 2.2 ± 0.2/0.34 ± 0.02 nm (right) at both sides and one linear row (marked by two parallel dashed lines) with a width/height of 1.9 ± 0.2/0.28 ± 0.02 nm at the middle. The inset of Figure 5c displays the filled-state image of the 9-NW, which clearly shows the 9-NWs grown epitaxially on the Si(110) surface. The mean NW width is broadened to 5.3 ± 0.2 nm and the typical height is increased to 340 ± 20 pm. The average pitch is enlarged to 6.3 ± 0.2 nm, similar to that of the parallel 6-NWs (i.e., 6.0 ± 0.2 nm). Obviously, the left-right asymmetry observed in the topography of the 9-NW is similar to that of the 6-NW. Moreover, the total width of both the right zigzag chain and the linear row in the 9-NW (i.e., 4.1 ± 0.2 nm) is close to that of the double zigzag chains of the 6-NW (i.e., 5.0 ± 0.2 nm). Therefore, the growth of the parallel-aligned 9-NWs follows the framework of the parallel array of the 6-NWs.

As seen in the inset of Figure 5a, the morphology of the substrate (the dark chain/row bundle marked by the dashed box at the left) is the same as that of the 9-NW (the bright chain/row bundle marked by the dashed box at the right). The topography profile of the substrate (Figure 5e) shows two nonequivalent zigzag chains with widths/heights of 1.4 ± 0.1/0.09 ± 0.005 nm (left) and 2.4 ± 0.1/0.16 ± 0.02 nm (right) at both sides and one linear row with a widths/heights of 1.8 ± 0.1/0.10 ± 0.01 nm in between. The widths of two chains and one row on the substrate are nearly equal to those of their counterparts in 9-NWs, respectively, but the heights of these two chains and one row on the substrate in Figure 5e are about half the heights of their counterparts in 9-NWs in Figure 5d. This result strongly indicates that the substrate can be regarded as a large-area parallel array consisting of 9-NWs with one-layer height (160 ± 20 pm). That is, the 9-NWs of two-layer height (340 ± 20 pm) exhibit a layer-by-layer growth mode. Multilayer NW growth is usually observed in the growth of other rare-earth silicide NWs [36].

### Growth mechanism

As clearly shown in Figures 2, 3, 4, and 5, Ce atoms preferentially adsorb on the long-range grating-like upper Si terraces of the Si(110)-16 × 2 surface to form well-ordered parallel arrays of 3-NWs at the first growth stage with 3-ML Ce deposition and then react concurrently with both periodic upper and lower terraces to produce mesoscopically ordered parallel arrays of 6-NWs at the second growth stage with 6-ML Ce deposition. When the Ce coverage is further increased to 9 ML, the growth of parallel-aligned 9-NWs follows the framework of the parallel array of the 6-NWs and exhibits a layer-by-layer growth mode to form multiple-layer NWs.

**Figure 6:**
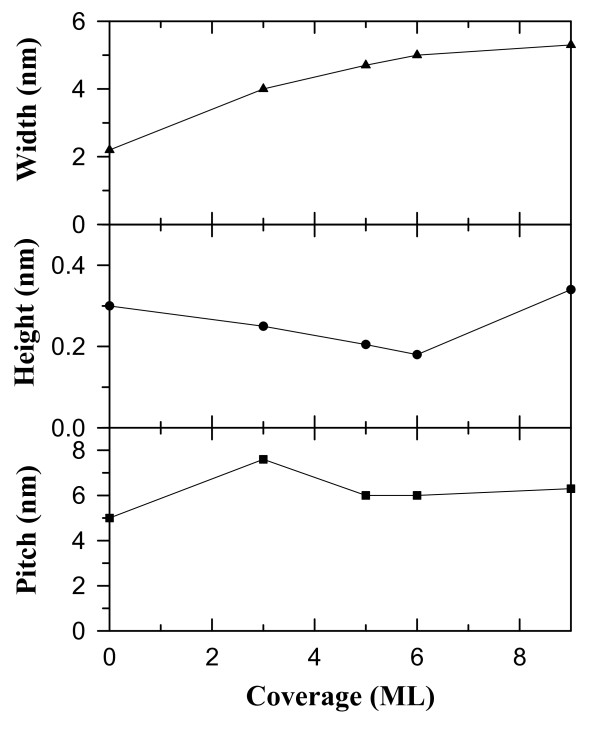
**The average dimensions of parallel CeSi**_***x ***_**NWs as functions of Ce coverage.**

**Figure 7:**
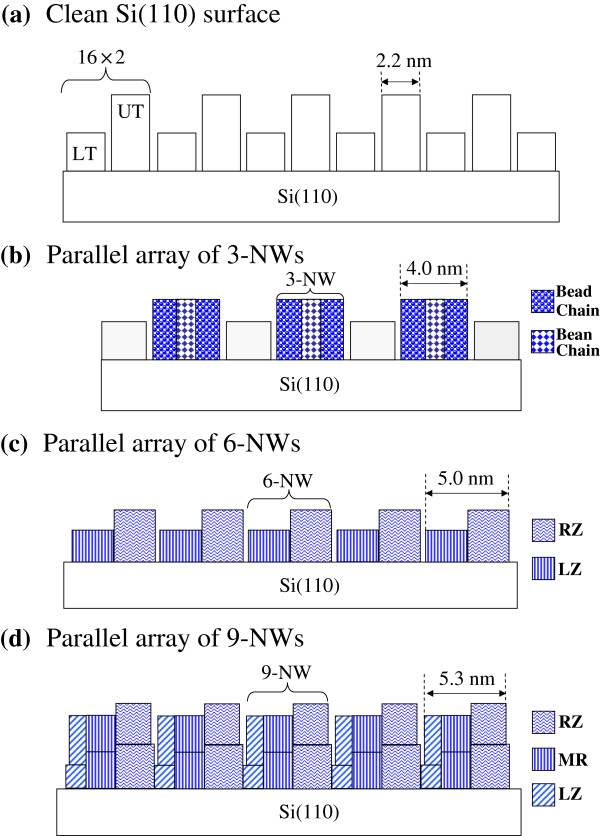
**Schematics of the growth evolution of parallel CeSi**_***x ***_**NW arrays on Si(110)-16 × 2 surfaces. (a)** Si(110)-16 × 2 surface. **(b, c, d)** Parallel arrays of 3-NW, 6-NW, and 9-NW. The upper and lower terraces on the Si(110) surface are labeled by UT and LT. The left and right zigzag chains in the 6-NWs and 9-NWs are labeled by LZ and RZ. The linear rows at the middle of the 9-NWs are labeled by MR.

Figure 6 presents the changes in the widths, heights, and pitches of various CeSi_*x*_ NWs formed at different Ce coverages. Due to the Si pentagon pairs with extra dangling bonds on the upper terraces of the 16 × 2 reconstruction, there is a considerable surface stress on the upper terraces to yield an electronically stable configuration. Consequently, the Si pentagon pairs on the upper terraces are likely to interact with Ce atoms upon the adsorption of Ce on the Si(110) surface, leading to the splitting of the Si pentagon pair on the upper terraces (Figure 2a). The NW width is thus broadened from 2.2 to 5.3 nm, which can be explained by the relaxation of the surface stress on the upper Si terrace upon Ce adsorption [37]. The stress relaxation also causes the pitch between the adjacent NWs to be increased from 5.0 to 7.6 nm, while after 3-ML deposition, the pitch is reduced to 6.3 nm due to the balance between the elastic energy in the terraces and the formation energy of 6-NWs. The apparent height of CeSi_*x*_ NW in the empty-state images is firstly decreased with the increase of Ce coverage and subsequently is increased due to the development of the second silicide layer on NWs. The gradual decrease of the NW height may be attributed to an inward vertical relaxation of Ce atoms upon additional Ce adsorption. The lengths of different CeSi_*x*_ NWs can exceed 1 μm, depending on the domain area of the 16 × 2 reconstruction. Figure 7 displays the schematic drawing to illustrate the growth evolution of the parallel CeSi_*x*_ NW arrays on Si(110)-16 × 2 surfaces with increasing Ce coverages. Additionally, the dual-polarity STM images clearly reveal that interchain coupling results in the formation of different registry-aligned chain bundles at the various growth stages of CeSi_*x*_ NWs. Thus, we have shown that the NW width and the interchain coupling can be adjusted systematically by varying the Ce coverage on Si(110).

### Prospects

The ability to grow mesoscopically ordered CeSi_*x*_ NW arrays on Si(110)-16 × 2 templates with atomic precision demonstrates that this template-directed 1D self-organization based on the single-domain Si(110)-16 × 2 surface can allow us to control accurately the growth and the electronic properties of individual NWs on an industrially reliable scale. Moreover, the massively parallel arrays of periodic and atomically identical CeSi_*x*_ NWs can provide an opportunity to understand precisely the exotic 1D physics of electrons in CeSi_*x*_ NWs by photoemission and photoabsorption spectroscopy study. Additionally, the high quality of these periodic arrays together with their easy fabrication render such supergratings as ideal nanotemplates for directing further deposition of functional units. For example, the subsequent deposition of O_2_ on these supergratings can fabricate different types of parallel arrays consisting of periodic CeO_2_ NWs for various catalytic, electronic, and optical applications.

## Conclusions

We have demonstrated a straightforward and efficient bottom-up nanofabrication for growing massively parallel arrays of highly periodic CeSi_*x*_ NWs on a single-domain Si(110)-16 × 2 surface with atomic precision. Three different types of massively parallel arrays, consisting of periodic and atomically identical CeSi_*x*_ NWs, are self-organized on the Si(110) surface at three Ce coverages of 3, 6 and 9 ML. The STM results show that the Si pentagon pairs serve as reactive nuclei for NW growth and account for the alignment of CeSi_*x*_ NWs on the periodic terraces of Si(110) surfaces. The self-organization mechanism of periodic CeSi_*x*_ NWs on Si(110) surfaces at different growth stages is presented. This natural template-directed self-organization of parallel CeSi_*x*_ NW arrays on Si(110) surfaces does not require an anisotropic lattice mismatch and can be applied to other RE metals. At the first growth stage, each 3-NW comprises double bead chains on two sides, separated by a bean chain. At the second growth stage, all periodic 6-NWs consist of double nonequivalent zigzag chains. At the third growth stage, parallel-aligned 9-NWs are composed of a bundle of double nonequivalent zigzag chains at two sides and one linear row in between. During the various growth stages, the interchain coupling result in the formation of different registry-aligned chains bundled within the individual CeSi_*x*_ NW. A variety of CeSi_*x*_ NWs with different chain bundles provides an opportunity for tailoring exotic electronic properties. The ability to precisely control the feature size and positions of periodic CeSi_*x*_ NWs within ±0.2 nm over a large area allows for wafer-scale integration into nanoelectronic devices.

## Competing interests

The authors declare that they have no competing interests.

## Authors' contributions

IHH designed the project of experiments and drafted the manuscript. YCL and YFT carried out the growth of CeSi_*x*_ nanowires and STM measurements. All authors read and approved the final manuscript.
